# A Rare Intruder: Neonatal Meningoencephalitis by *Edwardsiella tarda* Requiring Systemic and Intrathecal Antibiotics and Repeated Neurosurgery

**DOI:** 10.3390/antibiotics15010059

**Published:** 2026-01-05

**Authors:** Domenico Umberto De Rose, Ludovica Martini, Francesca Campi, Daniela Longo, Alessia Guarnera, Giulia Lucignani, Marta Conti, Alessandra Santisi, Carlotta Ginevra Nucci, Giacomo Esposito, Lorenza Romani, Paola Bernaschi, Bianca Maria Goffredo, Gianfranco Scarpelli, Laura Lancella, Andrea Dotta, Maria Paola Ronchetti

**Affiliations:** 1 Neonatal Intensive Care Unit, “Bambino Gesù” Children’s Hospital IRCCS, 00165 Rome, Italy; ludovica.martini@opbg.net (L.M.); alessandra.santisi@opbg.net (A.S.); andrea.dotta@opbg.net (A.D.); mariapaola.ronchetti@opbg.net (M.P.R.); 2 Functional and Interventional Neuroradiology Unit, “Bambino Gesù” Children’s Hospital IRCCS, 00165 Rome, Italy; daniela.longo@opbg.net (D.L.); giulia.lucignani@opbg.net (G.L.); 3 Neuroradiology Unit, NESMOS Department, “Sant’Andrea” Hospital, “Sapienza” University, 00189 Rome, Italy; guarneraalessia@gmail.com; 4 Faculty of Medicine and Surgery, UniCamillus, Saint Camillus International University of Health Sciences, 00131 Rome, Italy; 5 Child Neurology, Epilepsy and Movement Disorders, “Bambino Gesù” Children’s Hospital IRCCS, 00165 Rome, Italy; marta.conti@opbg.net; 6 Neurosurgery Unit, “Bambino Gesù” Children’s Hospital IRCCS, 00165 Rome, Italy; cginevra.nucci@opbg.net (C.G.N.); giacomo.esposito@opbg.net (G.E.); 7 Infectious Diseases Unit, “Bambino Gesù” Children’s Hospital IRCCS, 00165 Rome, Italy; lorenza.romani@opbg.net (L.R.); laura.lancella@opbg.net (L.L.); 8 Microbiology and Diagnostic Immunology Unit, “Bambino Gesù” Children’s Hospital IRCCS, 00165 Rome, Italy; paola.bernaschi@opbg.net; 9 Division of Metabolic Diseases and Drug Biology, “Bambino Gesù” Children’s Hospital IRCCS, 00165 Rome, Italy; biancamaria.goffredo@opbg.net; 10 Division of Neonatology and Neonatal Intensive Care Unit, “SS. Annunziata” Hospital, 87100 Cosenza, Italy; gianfranco_scarpelli@virgilio.it

**Keywords:** brain abscess, pregnancy, Enterobacterales, *Edwardsiella*, vertical transmission, neonatal sepsis, intrathecal antibiotics, review, case report

## Abstract

**Background/Objectives**: *Edwardsiella tarda* is a rare Gram-negative pathogen that uncommonly infects humans. Neonatal infections are extremely rare but often severe, with a high incidence of central nervous system (CNS) complications. **Case presentation**: We report a term neonate born via spontaneous vaginal delivery who developed systemic signs of infection within 18 h of life. Blood and cerebrospinal fluid (CSF) cultures grew *Edwardsiella tarda*. CSF analysis revealed severe meningoencephalitis. Maternal stool culture was also positive for *E. tarda*, suggesting vertical transmission. Despite initial systemic antibiotic therapy with ampicillin, gentamicin, and ceftriaxone, neuroimaging revealed progressive multifocal brain abscesses. The infant underwent a series of neurosurgical procedures, including bilateral drainage of abscesses, Rickham reservoir placement and ventriculoperitoneal shunting. A revised antibiotic regimen, including systemic meropenem and trimethoprim-sulfamethoxazole plus intrathecal gentamicin, was administered. At six months, the infant showed mild motor delay with lower limb hypertonia and was under close neurosurgical and developmental follow-up. **Methods**: We conducted a literature review of 12 published neonatal *E. tarda* infections, including our case. **Results**: Most infected infants presented within 72 h of life and exhibited CNS involvement. Mortality was 25%, and 44% of survivors experienced long-term neurologic sequelae. **Conclusions**: *Edwardsiella tarda* infection in neonates is rare but potentially devastating. Early suspicion, culture confirmation, aggressive antibiotic therapy, and multidisciplinary care, including neurosurgical management, are essential for improving outcomes.

## 1. Introduction

*Edwardsiella tarda* is a facultative anaerobic Gram-negative bacillus of the *Enterobacteriaceae* family, primarily known as an aquatic pathogen. Human infections with *E. tarda* are rare and typically manifest as gastroenteritis, wound infections, or soft tissue abscesses, most often in immunocompromised adults or in those with direct exposure to freshwater environments or raw aquatic food products [1,2,3]. In neonatal cases, published reports described a high rate of central nervous system (CNS) complications, including meningitis, ventriculitis, and multifocal brain abscesses [4,5,6,7,8,9].

Different reports have highlighted the likelihood of vertical transmission, with *E. tarda* isolated from maternal vaginal or stool samples [5,7], and intrauterine infection suspected in association with chorioamnionitis or premature rupture of membranes [6,10,11]. However, due to the rarity of this pathogen in humans and the lack of routine maternal screening, perinatal transmission may often go unrecognized.

We present a case of *E. tarda* meningoencephalitis with multifocal brain abscesses in a term neonate, successfully managed through prolonged multimodal therapy, including systemic and intrathecal antibiotics and serial neurosurgical interventions. To our knowledge, this represents one of the most severe neonatal CNS presentations caused by *E. tarda* described in the literature, underscoring the importance of early recognition, microbiologic confirmation, and coordinated multidisciplinary care.

## 2. Case Presentation

### 2.1. Edwardsiella Tarda Infection

We report the case of a term newborn (38 weeks + 6 days of gestation), appropriate for gestational age, born via spontaneous vaginal delivery with Apgar scores of 9 and 10 at 1 and 5 min, respectively. The pregnancy was uneventful, with negative vaginal-rectal swabs for Group B Streptococcus and no evidence of acute maternal TORCH infections. Notably, however, the mother had a history of recurrent episodes of gastroenteritis during pregnancy.

The neonate exhibited good initial cardiopulmonary adaptation, but at approximately 18 h of life developed signs of systemic illness, including low-grade fever, hypo-responsiveness, and cutaneous mottling. He was promptly transferred to the Neonatal Intensive Care Unit (NICU) at a regional hospital.

On admission, laboratory evaluation showed elevated inflammatory markers. Blood and cerebrospinal fluid (CSF) cultures were obtained, and empiric broad-spectrum antibiotic therapy was initiated with ampicillin (50 mg/kg every 8 h), gentamicin (5 mg/kg every 24 h), and ceftriaxone (100 mg/kg every 24 h). Culture results revealed *Edwardsiella tarda* in both blood and CSF, a rare Gram-negative facultative anaerobe, infrequently associated with neonatal infections. The antibiogram revealed that *E. tarda* was susceptible to all tested antibiotics, including ampicillin, ceftriaxone, meropenem, and trimethoprim-sulfamethoxazole (Table 1).

CSF analysis was significantly abnormal, consistent with bacterial meningoencephalitis: the cerebrospinal fluid appeared to be xanthochromic, with 17,948 white blood cells/mm^3^ (860 polymorphonuclear neutrophils and 140 lymphocytes), 10,000 red blood cells/mm^3^, immunoglobulins 179 mg/dL, and albumin 681 mg/dL. Maternal cultures were performed: stool culture was positive for *Edwardsiella tarda*, while microbiological analysis of breast milk tested negative. She declared no recent ingestion of raw fish during pregnancy. The patient was treated with triple antibiotic therapy consisting of gentamicin, ampicillin, and ceftriaxone. After three days, blood culture was already negative.

The neuroimaging examinations revealed extensive central nervous system involvement. Specifically, the baseline Computed Tomography (CT) showed diffuse white matter (WM) hypodensity and four heterogeneously hypodense lesions centered in the periventricular WM, at the level of the left and right frontal horns and the left and right ventricular trigones. A brain Magnetic Resonance Imaging (MRI) exam, limited by severe motion artifacts and performed without contrast injection, was obtained 14 days later. It confirmed the presence of the four lesions, which exhibited heterogeneous and marked diffusion restriction on Diffusion-Weighted Imaging (DWI) and perilesional WM hyperintensity on the T2 Weighted-Imaging (WI).

The clinical course was complicated by the onset of acute symptomatic seizures, for which the infant required antiepileptic treatment including phenobarbital and continuous midazolam infusion. Electroencephalographic (EEG) monitoring confirmed the presence of electroclinical and subsequently subclinical epileptic activity. Seizures were successfully controlled with levetiracetam monotherapy after tapering phenobarbital.

Due to the complexity and severity of the case, the infant was transferred to our III-level NICU at 20 days of life. Upon admission, he was in spontaneous breathing and receiving both enteral feeding via nasogastric tube and parenteral nutrition. All blood and CSF cultures obtained after transfer to our institution remained persistently negative.

**Figure 1:**
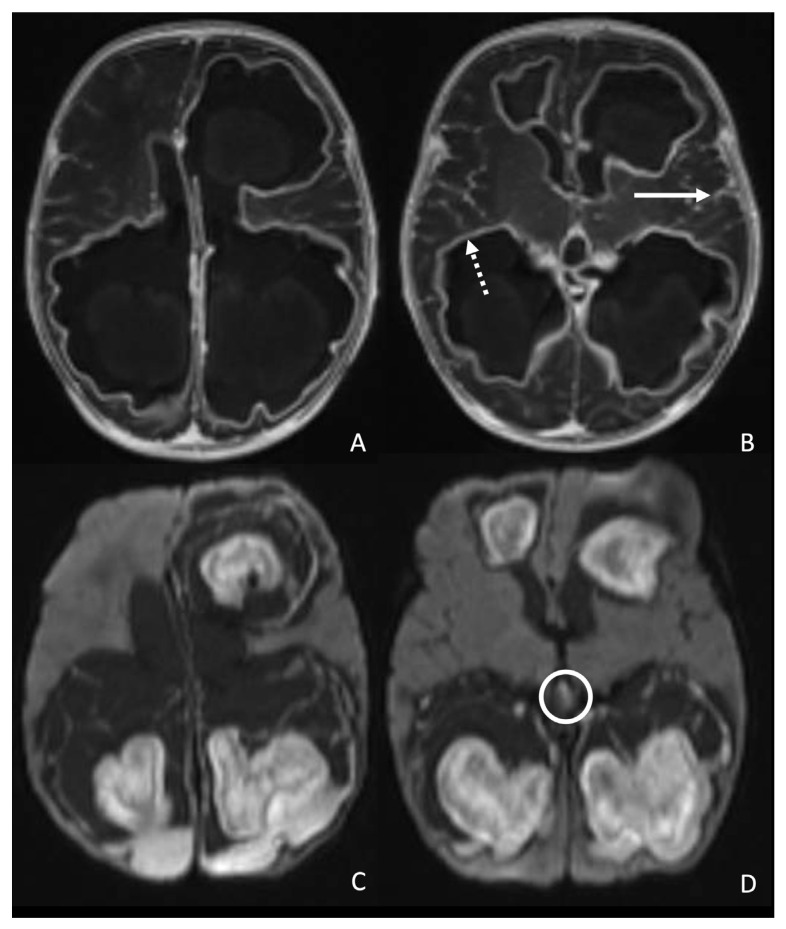
The figure illustrates the first two MRI scans performed at our institution, 2 days (**A**,**B**) and four days (**C**,**D**) after the initial MRI scan acquired at the birth center. The first MRI included a post-contrast 3D T1 MPRAGE (Magnetization Prepared Rapid Gradient Echo Imaging) sequence (**A**,**B**), which revealed four heterogeneous lesions with intense peripheral contrast enhancement located in the periventricular white matter, at the level of the left and right frontal horns and the left and right ventricular trigones (**A**,**B**). The lesions adjacent to the trigones and right frontal horn showed extensive communication with the ventricles (**A**). Additional findings included marked enhancement of the ependymal lining (dotted white arrow in (**B**)) and the leptomeninges (white arrow in (**B**)), consistent with meningoencephalitis. The second MRI, which included a Diffusion-Weighted Imaging (DWI) sequence (**C**,**D**), demonstrated heterogeneous diffusion restriction of the four abscesses and extensive ventricular septations (**C**,**D**). Also, it revealed abscess-like tissue within the third ventricle (white circle in (**D**)), supporting the suspicion of intraventricular abscess dissemination.

We performed a follow-up brain MRI two days after admission to our center. The four lesions located in the periventricular WM, at the level of the left and right frontal horns and the left and right ventricular trigones, demonstrated intense peripheral contrast enhancement on the post-contrast 3D T1 MPRAGE (Magnetization Prepared Rapid Gradient Echo Imaging) sequence. Associated findings included marked enhancement of the ependymal lining and leptomeninges, supporting the diagnosis of meningoencephalitis. Moreover, three abscesses exhibited extensive communication with the trigones and the right frontal horn of the ventricles, raising suspicion of intraventricular dissemination. This was confirmed on a subsequent brain MRI performed four days later, which showed heterogeneous diffusion-restriction of the four abscesses on DWI and extensive ventricular septa, and revealed abscess-like tissue in the third ventricle (Figure 1).

### 2.2. Neurosurgical Interventions

The patient underwent the first neurosurgical intervention at the age of 23 days. A left frontal cystic catheter connected to a 6 mm Rickham reservoir was placed under general anesthesia to drain one of left-brain abscesses. The procedure involved a precoronary left frontal incision, creation of a bone window, dural opening, and catheter insertion with aspiration of clear, citrine CSF. Given the lack of neuroradiological improvement, after 3 weeks, a right frontal intraventricular catheter connected to a subcutaneous Rickham reservoir was positioned via a small craniectomy under ultrasound guidance. During the same intervention, the previously implanted left frontal Rickham reservoir was mobilized and rotated to optimize its position. CSF samples showed xanthochromia, and fontanelle tension was normalized after drainage. Subsequently, after further 3 weeks, neuronavigated drainage of bilateral intracranial abscesses was performed through parieto-occipital craniotomies. Purulent material was gently aspirated from both abscess cavities, followed by irrigation, resulting in decompression of the fontanelle.

Following these procedures, at 2 months of life, the patient subsequently underwent a left-sided endoscopic septostomy and placement of a ventriculoperitoneal shunt with a programmable Hakim valve, initially set to 12 cmH_2_O, to address progressive post-infectious multicystic hydrocephalus.

### 2.3. Systemic and Intrathecal Antibiotic Treatment

At admission in our NICU, the previous antimicrobial regimen was suspended and replaced with meropenem (40 mg/kg every 8 h) and trimethoprim-sulfamethoxazole (7 mg/kg every 8 h). Meropenem was measured simultaneously in blood and cerebrospinal fluid (CSF), demonstrating adequate blood and CSF concentrations (4.5 mcg/mL and 1.41 mcg/mL, respectively, at approximately 7 h after administration). As meropenem is a time-dependent antibiotic, we decided to extend the infusion time to 4 h, compatible with the need to use the central venous catheter for the administration of other therapies. An improvement in CSF characteristics was achieved (a simultaneous reduction in the white blood cell counts and protein, together with an improvement in glycorrhachia), and any reduction in the size of the abscess lesions was described. Intrathecal therapy was initiated with Gentamicin (5 mg at a concentration of 1 mg/mL) due to concerns about the appearance of a fifth abscess in the third ventricle. The first dose of Gentamicin was administered through the right catheter to exclude an altered communication of the ventricular system; a cerebrospinal fluid (CSF) measurement was performed after 24 h from both catheters with the detection of coincident concentrations. This was indicative of good communication between the two sides and a homogeneous diffusion of the drug in the CSF (respectively, 31.85 mcg/mL in the right side and 35.78 mcg/mL in the left side 24 h after the first administration through the right catheter and, before the second administration). Beyond systemic antibiotic treatment, intrathecal gentamicin was administered through the reservoirs for seven days on alternating sides. Systemic antibiotic therapy was discontinued after 45 days. No renal, hepatic, hematologic, or electrolyte abnormalities attributable to antibiotic therapy were observed during systemic and intrathecal treatment.

Cultures of CSF and blood performed in our hospital remained persistently negative. Throughout this period, the patient received close monitoring, antibiotic therapy, and wound care, with imaging and neurological assessments guiding surgical timing and interventions.

Immunologic evaluation was conducted prior to routine immunizations before discharge. Results revealed mildly decreased IgA levels (age-appropriate), a slight inversion in the CD4/CD8 ratio, and normal lymphocyte subset distribution. Neutrophil oxidative burst testing and other immunoglobulin levels (IgG, IgM, IgE) were within normal limits.

### 2.4. Further Management and Follow-Up

Nutritional management included combined enteral and parenteral feeding, with gradual advancement of enteral nutrition following neurosurgical stabilization and resumption of intestinal peristalsis, discontinuing parenteral nutrition.

Ophthalmologic evaluation showed normal fundus oculi. Audiologic screening (Transient-Evoked Otoacoustic Emissions and Auditory Brainstem Response) demonstrated bilateral normal auditory function. Cardiac evaluation via echocardiography revealed no structural or functional abnormalities. Renal ultrasound, however, showed bilateral enlarged kidneys with reduced corticomedullary differentiation and mild hyperechogenicity of the medullary pyramids, warranting continued nephrological follow-up.

At the time of reporting, the infant remained clinically stable, with no further seizure activity and no new signs of systemic infection. He was receiving oral levetiracetam, maintaining adequate nutritional intake, and undergoing regular neurosurgical and neurological follow-up. At 6 months of age, axial tone was good, with prevalent hypertonia in the lower limbs. Motor activity was symmetrical, though tends to be jerky.

## 3. Materials and Methods

We performed a structured literature review of neonatal infections caused by *Edwardsiella tarda*. A MEDLINE (PubMed) search was conducted covering the period from 2000 to 28 July 2025. The search strategy combined Medical Subject Headings (MeSH) and free-text terms using the following Boolean expression: (“*Edwardsiella tarda*”) AND (“pregnancy” OR “maternal infection” OR “neonate” OR “newborn” OR “neonatal sepsis” OR “meningitis”). Reference lists of all included papers were hand-searched to identify additional relevant reports before 2000 (“snowball method”), including articles from 1968. No filters for article type were applied initially to avoid missing historical case descriptions referenced in modern publications.

Studies were included if they met all the following criteria: (1) human neonates (0–28 days) or perinatal infections with neonatal onset; (2) microbiological identification of *Edwardsiella tarda* from any neonatal or maternal sample; (3) full case description with extractable clinical data; (4) English-language publication (due to lack of certified translations). We have excluded cases published only in Japanese or other languages.

Two authors (D.U.D.R. and L.M.) independently reviewed all eligible articles and extracted predefined variables using a structured form. Discrepancies were resolved by consensus with a senior reviewer (M.P.R.). Extracted data included neonatal characteristics (gestational age, birthweight, mode of delivery, timing of symptom onset), neonatal clinical presentation (features of sepsis, neurological involvement, need for ventilation or intensive care), neonatal management (antibiotic regimens, duration of therapy, neurosurgical procedures, antibiotics-related adverse events), microbiological data (culture sites, antibiotic susceptibility patterns when available), maternal history (gastrointestinal symptoms, aquatic exposure, raw fish consumption, colonization with *E. tarda*), outcome (survival, neurological sequelae, need for shunting, or other complications).

Given the rarity of the pathogen and the heterogeneity of reporting, a descriptive, narrative synthesis was chosen. Quantitative pooling was not possible due to the variability in case reporting across decades. No statistical analyses were performed due to the small sample size and case-report nature of the data.

## 4. Results

Table 2 summarizes cases of *Edwardsiella tarda* infections during the neonatal period, published in English language [4,5,6,7,8,9,10,11,12,13]. A total of 12 neonatal infections were analyzed, spanning from 1968 to 2025.

Gestational ages ranged from 30 to 40 weeks, with birthweights ranging from very low birth weight to appropriate-for-gestational-age weight at term. Most neonates (11/12) were born by vaginal delivery, and only one required an emergency cesarean section due to fetal distress. Home birth was reported in one fatal case [12].

Onset of symptoms varied from within hours after birth [6,8] to as late as day 10 of life [8]. Clinical manifestations typically included lethargy, fever, sepsis, feeding difficulties, respiratory distress, and jaundice. Notably, CNS involvement was observed in 10/12 cases (83.3%), including meningitis, ventriculitis, seizures, cerebral edema, and brain abscesses. Three infants of ten (30%) presented with or developed hydrocephalus requiring ventriculoperitoneal shunting.

Maternal *E. tarda* colonization was demonstrated in 6/12 cases, through vaginal or stool cultures, supporting vertical transmission.

Antibiotic therapy was administered in all survivors, with initial regimens often including ampicillin and gentamicin, later escalated to cefotaxime, cefepime, meropenem, or trimethoprim-sulfamethoxazole. Treatment durations ranged from a few days to more than 6 weeks, especially in severe CNS cases. Our infant received intrathecal gentamicin in addition to systemic therapy.

Neurosurgical intervention was required in 4/12 cases, primarily for drainage of brain abscesses or hydrocephalus management. Three infants died [4,8,12], all within the first month of life. Among the nine survivors, four (44.4%) had long-term neurological sequelae, including cerebral palsy, hydrocephalus, and sensorineural hearing loss, while five (55.5%) showed normal or mildly delayed development on follow-up. Therefore, death or adverse neurodevelopmental outcomes were reported in seven of 12 patients (58.3%).

## 5. Discussion

*Edwardsiella tarda* is a rare but potentially severe pathogen in neonatal sepsis, with a high frequency of reported CNS involvement in the available neonatal cases [9,11]. The present review of 12 cases, including both historical and recent reports, reveals consistent patterns in clinical presentation, suspected transmission, and outcomes. Higashigawa et al. reviewed additional cases published in Japanese, which we excluded due to language barriers: the authors highlighted the habit of consuming raw fish as one of the reasons behind the numerous reports of *E. tarda* infections in Japan [11].

Most affected neonates were delivered vaginally, at or near term, and frequently had perinatal risk factors, including maternal gastrointestinal symptoms during pregnancy [4], contact with freshwater sources [5,7], or raw fish consumption [11]. Maternal colonization with *E. tarda* was microbiologically confirmed in different cases through stool or vaginal swabs [5,7], suggesting vertical or ascending transmission. In some cases, prolonged rupture of membranes or chorioamnionitis may have facilitated intrauterine infection [6,10,11]. Similarly, in our case, the recurrent episodes of gastroenteritis during pregnancy could have contributed.

One of the most striking findings across the series is the high frequency of early and extensive CNS involvement, reported in 10 of 12 cases. These ranged from meningitis and ventriculitis [6,7], to multiple brain abscesses requiring neurosurgical drainage [9]. Unlike common neonatal pathogens such as *E. coli* or Group-B Streptococcus, CNS involvement was common among the published *E. tarda* case reports, often necessitating aggressive antibiotic escalation and neurosurgical intervention. This marked neurotropism may be partly explained by specific virulence determinants of *Edwardsiella tarda*. Experimental studies have demonstrated the role of type III and type VI secretion systems in epithelial invasion, intracellular survival, and systemic dissemination. Such mechanisms may facilitate blood–brain barrier penetration, particularly in neonates with immature immune defenses [14,15]. In addition, *E. tarda*–induced cytotoxicity has been shown to depend on type III secretion system effectors and flagellin, further supporting its ability to cause tissue damage and invasive disease [16].

Empiric therapy often included ampicillin and gentamicin, but was frequently escalated to cefotaxime, meropenem, or trimethoprim-sulfamethoxazole due to CNS complications, such as in the case reported by Takeuchi’s group [9] and in our case. The use of intrathecal antibiotics, such as gentamicin administered via Rickham reservoirs, is rare in neonatal practice but was deemed necessary in our case due to the extent and complexity of the brain abscesses. In adult patients, including immunocompromised hosts, *E. tarda* infections are generally susceptible to beta-lactams, fluoroquinolones, and carbapenems, although disseminated and severe disease has been described [17]. While susceptibility patterns appear broadly comparable, neonatal infections are distinguished by earlier CNS involvement and a narrower therapeutic window.

Despite the organism’s general antibiotic susceptibility [4,5], mortality remains high when recognition or treatment is delayed. In three cases [4,8,12], neonates died within days due to overwhelming sepsis or neurologic damage. Among the nine surviving infants, four developed lasting neurologic sequelae, including cerebral palsy [7,10], sensorineural hearing loss [5], or post-infectious hydrocephalus with motor impairment, such as in our case. On the other hand, five cases (including one requiring multiple surgeries reported by Takeuchi et al. [9]) demonstrated normal or near-normal neurodevelopmental outcomes. Beyond host immaturity, disease severity in neonatal *E. tarda* infections may also reflect pathogen-specific virulence factors, as highlighted in broader analyses of Enterobacterales causing neonatal sepsis [18]. Severe systemic *Edwardsiella tarda* infections with bacteremia have also been described in adult populations, particularly in Japan, with reported high morbidity and mortality, reinforcing the pathogenic potential of this organism even outside neonatal settings [19].

Concerning limitations, while our case contributes to the growing body of literature on *Edwardsiella tarda* neonatal infections, broader conclusions about management strategies, prognosis, or epidemiology require larger case series or multicenter studies. Although maternal stool cultures were positive for *E. tarda*, the precise mode and timing of transmission (e.g., ascending intrauterine infection vs. peripartum exposure) could not be definitively established. Environmental or dietary sources of maternal infection were not microbiologically confirmed. At the time of reporting, only early neurodevelopmental follow-up (up to 6 months of age) was available. Longer follow-up is necessary to assess the full extent of neurological sequelae, especially given the severity of brain injury and need for repeated neurosurgical interventions. Although cultures confirmed *E. tarda* in both infant and mother, molecular techniques such as whole-genome sequencing were not performed to establish strain identity or assess virulence factors. Indeed, *E. tarda* uses virulence factors that include type III and type VI secretion systems, quorum sensing, two-component systems, and exoenzymes that facilitate host invasion and intracellular survival [15]. Finally, the decision to pursue multiple invasive neurosurgical procedures, including catheter placement and abscess drainage, was based on local institutional expertise. Management of multiple brain abscesses in neonates remains challenging and often requires individualized, multidisciplinary strategies, as supported by broader evidence on CNS abscess management [20].

From a broader perspective, *E. tarda* represents a pathogen at the human–animal–environment interface, underscoring the relevance of a One Health framework in understanding maternal exposure and neonatal infections. The organism can be distributed in aquatic environments and colonizes a variety of animals, particularly fish and reptiles, facilitating zoonotic transmission through environmental exposure, food handling, or dietary habits. In this context, maternal exposure may act as a critical intermediary between environmental reservoirs and the neonate, especially during pregnancy, when gastrointestinal colonization or subclinical infection can precede vertical or ascending transmission [21].

## 6. Conclusions

In conclusion, although rare, *Edwardsiella tarda* infection should be considered in neonatal sepsis with CNS involvement, especially when there is a history of maternal gastrointestinal illness, aquatic exposure, or perinatal inflammation. Although the published cases reported maternal gastrointestinal symptoms or colonization, the limited number and heterogeneity of available reports do not allow any recommendation for routine maternal screening. These findings should therefore be interpreted as descriptive observations rather than generalizable clinical indications.

Neurosurgical procedures were performed in severe presentations with abscesses or hydrocephalus, but the evidence base remains too limited to draw generalizable practice recommendations. Decisions should be tailored to each infant’s clinical course and local expertise.

Taken together, these considerations underscore the need for individualized care; however, when *E. tarda* is suspected, timely diagnostic confirmation becomes crucial, and a multidisciplinary treatment (involving neonatologists/NICU team, microbiologists, pediatric infectious diseases specialists, neurologists, neuroradiologists, neurosurgeons, clinical pharmacologists, and neurorehabilitation specialists) are essential to improve prognosis and reduce neurologic morbidity.

## Figures and Tables

**Table 1:** Antibiogram of the *Edwardsiella tarda* strain isolated from blood and cerebrospinal fluid cultures.

Antibiogram	RSI	MIC	Break Point MIC
Amikacin	S	4	8
Amoxicillin/clavulanic acid		≤2	
Cefepime	S	≤0.12	
Ceftazidime	S	≤0.12	1
Colistin	R	16	2
Ertapenem	S	≤0.12	0.5
Gentamicin	S	≤1	2
Meropenem	S	≤0.12	2
Trimethoprim/sulfamethoxazole	S	≤20	40
Piperacillin/tazobactam	S	≤4	8
Tigecycline		≤0.5	
Ceftazidime/avibactam	S	≤0.25	
Ceftozolane/tazobactam	S	≤0.25	
Tobramycin	S	≤0.5	2
Imipenem/relebactam	S	0.12	
Meropenem/vaborbactam	S	≤0.06	

**Table 2:** Literature reports of neonatal infections by *Edwardsiella tarda* compared to our case.

Author, Year	Gestational Age (weeks)/Birthweight (Grams)	Onset	Delivery Type	Neonatal Clinical Features	Neonatal Management	Positive Cultures for *E. tarda*	Maternal History	Neurological Outcome of the Child
Okubadejo and Alausa, 1968 [8]	Term/NA	Day 10	Vaginal	Poor suckling, loss of weight, fever, with fatal septicemia and meningitis/At autopsy the brain was soft, with a collection of 50 mL of mucopurulent fluid over the surface, meningitis and hydrocephalus	Penicillin, Chloramphenicol, sulphadiazine. After 2 days of unsettled fever, streptomycin was added. Then colistin was given for fever resurgence. Not reported antibiotics-related adverse events.	Positive CSF culture for *E. tarda*	Unregistered	Death after 4 weeks of hospitalization
Vohra et al., 1988 [4]	33 wks/1820 g	Day 3	Vaginal	Jaundice, lethargy, hypotonus, sudden apneic spell with cyanosis and bradycardia, hypotension requiring resuscitation/At autopsy the lungs were congested with petechial hemorrhage; hemorrhage was seen in the adrenals, cerebellum and stomach mucosa. There were meningeal congestion and intraventricular hemorrhage with extension into the white matter.	One dose of aqueous penicillin G and gentamycin. Not reported antibiotics-related adverse events.	*E. tarda* isolated in Columbia broth from post-mortem spinal fluid, blood, brain and heart; the organism was susceptible in vitro to ampicillin, cefazolin, cefoxitin, tetracycline, gentamicin, amikacin and trimethoprim-sulfamethoxazole	Hypertension during the third trimester and self-limited diarrhea 5 days before delivery; Amniotic fluid was clear	Death after 3 h since cardiorespiratory symptoms
Mowbray et al., 2003 [5]	Term/NA	Day 6	Vaginal	Lethargy, poor feeding, sepsis, jaundice	Ampicillin + gentamicin (5 d) → ampicillin alone (5 d); NICU support care. Not reported antibiotics-related adverse events.	Blood (infant), stool & vaginal swab (mother); identical by rep-PCR	Immersion in freshwater lake at 6 months gestation; maternal swabs for *E. tarda*	Right-sided profound hearing loss
Takeuchi et al., 2009 [9]	Term/NA	~6 h	Vaginal	High fever (38.9 °C), vomiting, moaning, low oxygen saturation (80%), feeding refusal; multiple brain abscesses and cerebritis requiring external drainage of the abscesses; post-encephalitis hydrocephalus requiring ventriculoperitoneal shunt	Cefotaxime + Ampicillin (11 days) → Meropenem (38 days). Not reported antibiotics-related adverse events.	Likely intrauterine infection; no maternal vaginal culture evidence; *E. tarda* identified from the cultured pus of brain abscesses (infant)	No raw fish consumption; no association with marine or water immersion	Normal developmental milestones, attending kindergarten
Hashavya et al., 2011 [7]	37 wks/3245 g	~12 h	Vaginal	Sepsis, seizures, cerebral edema, cystic brain lesions	Gentamicin + cefotaxime, phenobarbital. Not reported antibiotics-related adverse events.	Blood (infant), vaginal swab (mother)	Fever, foul-smelling amniotic fluid, chorioamnionitis; swam in Sea of Galilee	Severe cerebral palsy with spastic quadriplegia, microcephaly
Kadam, 2013 [12]	30 wks/1100 g	Day 4	Vaginal (home birth)	Sepsis, omphalitis, altered sensorium, respiratory failure	Ceftizoxime + amikacin → meropenem. Not reported antibiotics-related adverse events.	Blood	Unregistered, no antenatal care; cow dung on umbilicus; goat/cow milk feeding	Death within 24 h
Miyazawa et al., 2018 [10]	40 wks/3173 g	Birth	Cesarean (emergency)	Hypoxic–ischemic encephalopathy, multicystic encephalomalacia	NICU support care; therapeutic hypothermia; meropenem → ceftazidime. Not reported antibiotics-related adverse events.	Skin, pharynx, gastric fluid (infant); wound & hematoma (mother)	Chorioamnionitis, septic shock, wound abscess with *E. tarda*	Cerebral palsy (follow-up)
Egashira et al., 2020 [13]	27 wks/1014 g	Day 0	Vaginal	Sepsis, pulmonary hemorrhage, cerebral hemorrhage	Ampicillin/sulbactam (10 d) + cefotaxime (21 d), intravenous immunoglobulins. Not reported antibiotics-related adverse events.	Cord blood, amniotic fluid, maternal blood, nasal swab	Fever/chills before delivery; septicemia diagnosed; no raw fish exposure	Post-hemorrhagic cyst; no palsy
Higashigawa et al., 2023 [11]	37 wks/NA	/NA	Vaginal	Fever, jaundice, poor suckling, intracranial hemorrhage (intraventricular and subarachnoid hemorrhage), meningitis (CSF: 4773 WBCs/µL, 54 mg/dL protein, 60 mg/dl glucose)	Ampicillin/cloxacillin and cefotaxime for 3 weeks. Not reported antibiotics-related adverse events.	Detected via broad-range PCR targeting 16S rRNA gene and Basic Local Alignment Search Tool (BLAST) analysis; conventional cultures were negative	History of eating raw sweetfish (ayu) at 34 weeks of gestation; mild diarrhea 1 day before delivery (stool culture not performed); vaginal cultures negative for Streptococcus agalactiae, with premature rupture of membranes for 34 h	No neurological sequelae
Geibel et al., 2024 [6]	Term/NA	Day 3	Vaginal	Sepsis, seizures, meningitis, ventriculitis, brain abscess, hydrocephalus, venous thrombosis	Ampicillin + gentamicin → vancomycin + cefepime, abscess drainage, shunt, anticoagulation, seizure control. Not reported antibiotics-related adverse events.	Blood, CSF, middle ear fluid	Maternal endometritis; 12 h rupture of membranes; 4 fish tanks at home	Mild motor delay, ventriculomegaly at 2 yrs
De Rose et al., 2025 (present study)	38 wks/NA	~18 h	Vaginal	Sepsis, severe meningoencephalitis, brain abscesses requiring 3 neurosurgery interventions and shunt	Initial: Ampicillin + gentamicin + ceftriaxone → Meropenem + trimethoprim-sulfamethoxazole; intrathecal gentamicin (about 6 weeks). Not reported antibiotics-related adverse events.	Blood + CSF (infant), stool (mother)	Recurrent gastroenteritis in pregnancy; positive maternal stool for *E. tarda*	Ongoing follow-up: mild motor delay with lower limb hypertonia at 6 months; post-infectious hydrocephalus

## Data Availability

All data supporting the findings of this case report are included within the manuscript. Additional details can be provided by the corresponding authors upon reasonable request.

## Abbreviations

The following abbreviations are used in this manuscript:

ABR	Auditory Brainstem Response
CNS	Central Nervous System
CSF	Cerebrospinal Fluid
CT	Computed Tomography
DWI	Diffusion-Weighted Imaging
EEG	Electroencephalography
IgA	Immunoglobulin A
IgE	Immunoglobulin E
IgG	Immunoglobulin G
IgM	Immunoglobulin M
MRI	Magnetic Resonance Imaging
MPRAGE	Magnetization Prepared Rapid Gradient Echo
NICU	Neonatal Intensive Care Unit
PCR	Polymerase Chain Reaction
PMN	Polymorphonuclear Neutrophils
rRNA	Ribosomal Ribonucleic Acid
T2-WI	T2-Weighted Imaging
TEOAE	Transient-Evoked Otoacoustic Emissions
WM	White Matter

## References

[1] Janda J.M., Abbott S.L. (1993). Infections associated with the genus Edwardsiella: The role of *Edwardsiella tarda* in human disease. Clin. Infect. Dis..

[2] Chida R., Iio K., Kaizuka H., Hirai Y., Ishida Y., Yamanaka G. (2022). Chronic Diarrhea Associated with *Edwardsiella tarda* Gastroenteritis: A Case Report and Literature Review. Pediatr. Infect. Dis. J..

[3] Hasegawa K., Kenya M., Suzuki K., Ogawa Y. (2022). Characteristics and prognosis of patients with *Edwardsiella tarda* bacteremia at a single institution, Japan, 2005–2022. Ann. Clin. Microbiol. Antimicrob..

[4] Vohra K., Torrijos E., Jhaveri R., Gordon H. (1988). Neonatal sepsis and meningitis caused by *Edwardsiella tarda*. Pediatr. Infect. Dis. J..

[5] Mowbray E.E., Buck G., Humbaugh K.E., Marshall G.S. (2003). Maternal colonization and neonatal sepsis caused by *Edwardsiella tarda*. Pediatrics.

[6] Geibel E.M., Pearce M.R., Zabrocki L., Thompson C. (2024). Neonatal sepsis with meningitis, ventriculitis and brain abscess caused by *Edwardsiella tarda*. BMJ Case Rep..

[7] Hashavya S., Averbuch D., Berger I., Ofek-Shlomai N., Pitashny M., Hidalgo C., Ergaz Z. (2011). Neonatal sepsis following maternal amnionitis by *Edwardsiella tarda*: A case report and a review of the literature. Eur. J. Pediatr..

[8] Okubadejo O.A., Alausa K.O. (1968). Neonatal Meningitis Caused by *Edwardsiella tarda*. BMJ.

[9] Takeuchi H., Fujita Y., Ogawa H., Shiomi K., Toyokawa Y., Yamamoto T., Furukawa T., Ebisu T. (2009). Multiple brain abscesses in neonate caused by *Edwardsiella tarda*-case report. Neurol. Med. Chir..

[10] Miyazawa Y., Murakami K., Kizaki Y., Itaya Y., Takai Y., Seki H. (2018). Maternal peripartum septic shock caused by intrauterine infection with *Edwardsiella tarda*: A case report and review of the literature. J. Obstet. Gynaecol. Res..

[11] Higashigawa M., Ito M., Nashida Y. (2023). *Edwardsiella tarda* is an Important Causative Agent of Maternal-Fetal Infections in Pregnant Women: A Case Report and Japanese Literature Review. Jpn. J. Infect. Dis..

[12] Kadam S.D. (2013). *Edwardsiella tarda*—A case report. Indian J. Pediatr..

[13] Egashira M., Higuchi N., Shichijo A., Egashira T., Takayanagi T. (2020). Early-onset *Edwardsiella tarda* septicemia in an extremely preterm infant. Pediatr. Int..

[14] Tan Y.P., Zheng J., Tung S.L., Rosenshine I., Leung K.Y. (2005). Role of type III secretion in *Edwardsiella tarda* virulence. Microbiology.

[15] Leung K.Y., Siame B.A., Tenkink B.J., Noort R.J., Mok Y.K. (2012). *Edwardsiella tarda*—Virulence mechanisms of an emerging gastroenteritis pathogen. Microbes Infect..

[16] Xie H.X., Lu J.F., Rolhion N., Holden D.W., Nie P., Zhou Y., Yu X.J. (2014). *Edwardsiella tarda*-Induced cytotoxicity depends on its type III secretion system and flagellin. Infect. Immun..

[17] An L., Chan J.L., Nguyen M., Yang S., Deville J.G. (2023). Case Report: Disseminated *Edwardsiella tarda* infection in an immunocompromised patient. Front. Cell. Infect. Microbiol..

[18] Barcellini L., Ricci G., Bresesti I., Piazza A., Comandatore F., Sharland M., Zuccotti G.V., Folgori L. (2022). The Role of Virulence Factors in Neonatal Sepsis Caused by Enterobacterales: A Systematic Review. Int. J. Mol. Sci..

[19] Kamiyama S., Kuriyama A., Hashimoto T. (2019). *Edwardsiella tarda* Bacteremia, Okayama, Japan, 2005–2016. Emerg. Infect. Dis..

[20] Brouwer M.C., Tunkel A.R., McKhann G.M., van de Beek D. (2014). Brain abscess. N. Engl. J. Med..

[21] Schiller A., Saidouni A., Mahrous H., Elhakim M., Elkholy A. (2025). One Health best practices for addressing health threats at the human-animal-environment interface, with focus on the Eastern Mediterranean Region. Trans. R. Soc. Trop. Med. Hyg..

